# Incidence Rates and Time Trends of Skin Cancer in Golestan Province, Northeastern Iran, 2005-2018

**DOI:** 10.34172/aim.28801

**Published:** 2024-05-15

**Authors:** Majid Mehri, Mina Karazhian, Arash Nikyar, Romina Mehri, Ali Bagheri, Mahnaz Akbari, Gholamreza Roshandel, Mehrdad Teimoorian

**Affiliations:** ^1^Department of Internal Medicine, School of Medicine, Golestan University of Medical Sciences, Gorgan, Iran; ^2^Golestan Research Center of Gastroenterology and Hepatology, Golestan University of Medical Sciences, Gorgan, Iran; ^3^Department of Research and Technology, Golestan University of Medical Sciences, Gorgan, Iran; ^4^Deputy of Public Health, Golestan University of Medical Sciences, Gorgan, Iran; ^5^Deputy of Treatment, Golestan University of Medical Sciences, Gorgan, Iran; ^6^Stem Cell Research Center, Golestan University of Medical Sciences, Gorgan, Iran

**Keywords:** Epidemiology, Melanoma, Neoplasms, Skin cancer, Squamous cell

## Abstract

**Background:**

Given the significant occurrence of skin cancer in the Middle East and the existing research gap concerning its incidence and trends, this research aimed to study the epidemiology and trend changes of skin cancer in the Golestan province, Northeastern Iran.

**Methods:**

The Golestan Population-based Cancer Registry’s (GPCR’s) data bank was utilized to gather information on confirmed skin cancer cases in the province during 2005–2018. We used Poisson regression analysis for comparing incidence rates between groups. *P* values less than 0.05 were considered statistically significant.

**Results:**

Of 1690 patients (mean age: 62.05±15.83 years), most were male (60.1%) and resided in urban areas (61.5%). The age-standardized rate (ASR) of non-melanoma and melanoma skin cancer was 8.49 and 0.56 per 100000 persons-year, respectively. A notably higher ASR for non-melanoma skin cancer (NMSC) was observed in men (ASR: 10.60; 95% CI: 9.91-11.29) (*P*<0.01) and urban residents (ASR: 10.19; 95% CI: 9.52-10.82) (*P*<0.01). There was no significant difference in the ASR of melanoma skin cancer based on gender (*P*=0.24) and place of residence (*P*=0.48). The incidence trend of melanoma (estimated annual percent change [EAPC]: -3.28; 95% CI: -18.54 to 14.83) and NMSC (EAPC: 0.39; 95% CI: -3.99 to 4.97) did not differ significantly.

**Conclusion:**

During the 14-year study period, the ASR of both types of skin cancer exhibited a consistent pattern, except for NMSC, which showed higher rates among men and urban residents. This should be taken into consideration when formulating preventive and control strategies in the study area.

## Introduction

 Cancer is the leading cause of death in developed countries and the second leading cause in developing countries.^1,2^ Iran is a developing nation located in the Middle East, possessing a unique strategic location.^3^ In recent years, Iran has undergone substantial industry development and modernization, which has been accompanied by changes in the lifestyle and environment; these changes have affected the epidemiological patterns of various malignancies in the country such that cancer has become the second most common chronic non-communicable disease and the third most common cause of death following heart diseases and road accidents.^4,5^

 Despite the decrease in the overall rate of cancers in recent years, the incidence of skin cancers has increased by 3%–5% every year in the past two decades.^6^ As one of the most common malignancies in the world, skin cancers are usually classified into two types: melanoma and non-melanoma. The two most common types of non-melanoma skin cancer (NMSC) are basal cell carcinoma (BCC) and squamous cell carcinoma (SCC), which are often curable and of great epidemiological and clinical importance.^7,8^

 In 2017, melanoma was the fifth most common cancer in men and the sixth most in women in the United States. Although the incidence of melanoma is much lower than that of BCC or SCC, it has a much poorer prognosis.^9^ At the same time, NMSC accounts for at least 80% of all skin cancer cases, making it the most commonly diagnosed cancer.^10^ Based on a report by the World Health Organization (WHO), between 2 and 3 million NMSCs occur in the world every year. These cancers are anticipated as an important factor affecting the global burden of diseases in the coming decades.^11^

 In a study by the International Agency for Research on Cancer (IARC) in 2018, the estimated number of new cases (incidence) and age-standardized incidence rates (ASRs) of NMSCs were 13.7 and 10.1, respectively, while the incidence rate and ASR of in melanoma skin cancers were 3.8 and 3.1, respectively. In addition, the highest and lowest incidence rates were observed in North America (653.8 per 100 000 people) and Africa (60.1 per 100 000 people), respectively. According to this study, the incidence rates of non-melanoma and melanoma skin cancers in Iran were 3.7 and 0.61 per 100 000 people, respectively.^12^

 In a study on the epidemiology of skin cancer in Iran, the highest ASRs were observed in the provinces of Fars, Khuzestan, Bushehr, and Hormozgan. In addition, the overall prevalence of skin cancers in Iran is higher in men than women.^13^ The difference in the pattern of skin cancer occurrence in different geographical areas is mainly due to the difference in exposure to risk factors. These risk factors in NMSCs and some melanoma skin cancers include sunlight exposure, occupational exposure, ultraviolet exposure, pigmentary traits (red hair, fair skin, lack of tanning ability, and propensity to freckle), certain genetic disorders (including ocular albinism, Ferguson-Smith syndrome, and xeroderma pigmentosum), some diseases (including lichen planus, chronic discoid lupus erythematosus, and lichen sclerosus), and immunosuppression.^14,15^ It has been stated that immunodeficiency, especially in solid organ transplant recipients, leads to a 10-16-fold increased risk of BCC and a 65-250-fold increased risk of SCC compared with the general population.^7,16^

 According to the literature, infection with human papillomavirus (HPV), particularly beta-HPV, is associated with development of skin cancers, especially SCC. Therefore, close monitoring of patients with HPV infection for skin cancers is recommended.^17^ Saeidian et al developed a computational pipeline to detect genetic mutations and viral skin infections simultaneously, which could be valuable for diagnosing patients with viral dermatoses, including those caused by precancerous HPV infection.^18^

 In the United States, the annual cost of treating melanoma has grown faster than the costs for all cancers combined.^19^ The annual expenses for treating newly diagnosed melanoma cases are projected to rise substantially from $457 million in 2011 to $1.6 billion in 2030. This increase is attributed to the growing incidence rates, aging population, risky behaviors, and advancements in targeted therapies.^20^ Skin cancer is the third most frequent cancer in the Golestan province, Iran, preceded only by breast and colorectal cancers.^21^ A study conducted in 2004 on men only found that the incidence of skin cancer was 13.23 in this province, while the highest rate was reported in individuals aged 80-84 years.^22^ Despite the significant impact of skin cancers on the physical, emotional, and financial well-being of affected patients, there has been a lack of consistent investigation into the epidemiology of these cancers. Furthermore, monitoring the epidemiology of skin cancers is crucial for early detection, risk assessment, prevention, resource allocation, and advancing research in the field, ultimately contributing to better patient care and reduced skin cancer-related morbidity and mortality. Given the importance of skin cancers and the lack of sufficient epidemiological data in this region, this study was conducted to determine the incidence rate and temporal trends in skin cancer epidemiology in the Golestan province in the years 2005–2018.

## Materials and Methods

 A comprehensive analysis was carried out in the Golestan province, examining all documented occurrences of skin cancer between 2005 and 2018. After obtaining ethical approval from the Ethics Committee of the Golestan University of Medical Sciences (ethical approval code: IR.GOUMS.REC.384/1401), data of patients with skin cancer were retrieved from the databank of the Golestan Population-based Cancer Registry (GPCR), which records all data related to cancer cases after pathologic diagnosis. The GPCR is a high-quality cancer registry with more than 15 years of experiences. The standard operating procedure of the GPCR has been previously described in details (Ref: 29306787). Cancer-type coding was based on the International Classification of Diseases, Tenth Revision (ICD-10), and ICD-O-3 classification systems. In this study, patients with the code (ICD-10 C43-C44/melanoma and non-melanoma) were selected and included in the study. Data analysis was performed after data preparation and quality control. In addition, the percentage of cases that were diagnosed based on microscopic verification (MV%) of a tissue specimen or death certificate was also determined for each type of skin cancer.

###  Statistical Analysis

 The data collected was inputted into the SPSS software (version 16) and subsequently analyzed using descriptive statistics such as mean, standard deviation (SD), frequencies, and percentages. The incidence of cancers in the studied period was calculated based on the population of the province in the given years. In addition, the ASRs with 95% confidence intervals (95% CIs) were calculated by gender, type of cancer, and place of residence (city/village) using direct standardization method based on the world standard population. We used the 18-group World Standard Population (0-4, 5-9, …, ≥ 85) for standardization of incidence rates. All rates are reported per 100 000 persons-year. Poisson regression models were used to compare the incidence rates across genders and places of residence. *P *values less than 0.05 were considered statistically significant. The estimated annual percent change (EAPC) with 95% CI was calculated for assessing time trends in incidence rates of skin cancer. The statistical inference for time trend in incidence rate was made by estimated 95% CI of EAPC. If the 95% CI of EAPC does not contain the value 0, it is considered as a statistically significant trend. Finally, incidence time-trend graphs were drawn according to each of the aforementioned variables.

## Results

 Overall, 1690 new skin cancer cases were registered in the GPCR during 2005-2018. The mean age (SD) of the patients was 62.05 (15.83) years. The majority of the cases were male (60.1%) and living in urban areas (61.5%). Of 1,690 cases, 1581 (93.55%) were non-melanoma and 109 (6.45%) were melanoma cases (Table 1). The mean age of patients with NMSC (62.34 ± 15.70 years) was higher than that of patients with melanoma (57.91 ± 17.03 years).

**Table 1:** Number, Crude Rate, ASR, and 95% CI of ASR for Melanoma and NMSCs in the Golestan Province (Iran) During 2005–2018

**Variable**	**Non-melanoma**					**Melanoma**				
	**Number**	**Crude Rate**	**ASR**	**ASR_L**	**ASR_U**	**Number**	**Crude Rate**	**ASR**	**ASR_L**	**ASR_U**
Total population	1581	6.4	8.49	8.06	8.92	109	0.44	0.56	0.44	0.68
Gender										
Male	965	7.81	10.6	9.91	11.29	60	0.49	0.63	0.47	0.79
Female	616	4.98	6.45	5.92	6.98	49	0.40	0.49	0.35	0.63
Place of residence										
Urban	986	7.76	10.19	9.52	10.86	54	0.42	0.55	0.39	0.71
Rural	595	4.96	6.68	6.13	7.23	55	0.46	0.58	0.42	0.74

ASR, age-standardized rate.

 All melanoma cases and 95.95% of non-melanoma cases were diagnosed by the histopathological method (100% vs. 95.95% MV%). Fifteen cases (0.95%) of non-melanoma cancer were diagnosed by the death certificate only (DCO) method and the remaining 49 cases (3.10%) were diagnosed by clinical/paraclinical methods.

 Non-melanoma and melanoma skin cancers exhibited different ASRs with values of 8.49 and 0.56 per 100 000 person-year, respectively. The ASR of NMSC was significantly higher in males (ASR = 10.60; 95% CI: 9.91-11.29) than females (ASR = 6.45; 95% CI: 5.92-6.98) (*P* < 0.01). Moreover, the ASR of NMSC was significantly higher in urban areas (ASR = 10.19; 95% CI: 9.52-10.86) than rural areas (ASR = 6.68; 95% CI: 6.13-7.23) (*P* < 0.01). The ASR of melanoma did not show any notable variance with respect to gender (*P* = 0.24) or place of residence (*P* = 0.48).

**Figure 1:**
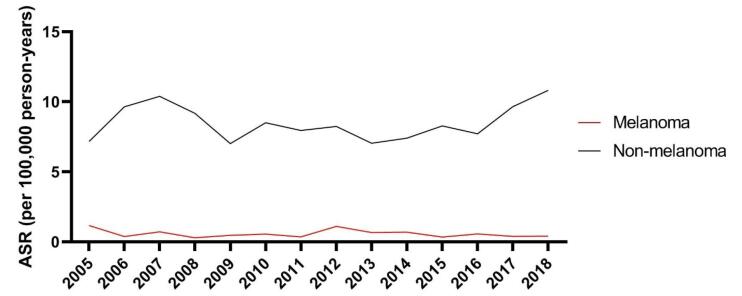
Comparison of the ASR (Per 100 000 Persons-Year) of Melanoma and Non-melanoma Skin Cancers According to the Year of Diagnosis

 There were no significant trends in incidence rates of melanoma (EAPC = -3.28; 95% CI: -18.54 to 14.83) and non-melanoma (EAPC = 0.39; 95% CI: -3.99 to 4.97) skin cancers (Figure 1).

**Figure 2:**
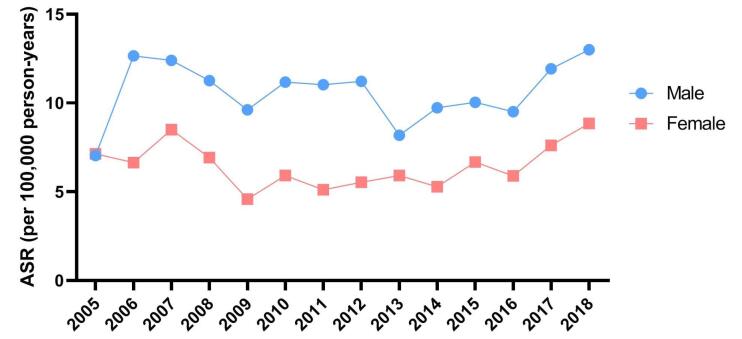
Time Trends of ASR (Per 100 000 Persons-Year) of Non-melanoma Skin Cancers by Gender

 As illustrated in Figure 2, there was no notable difference in the temporal pattern of NMSC incidence between males (EAPC = 0.55; 95% CI: -3.38 to 4.64) and females (EAPC = 0.39; 95% CI: -4.61 to 5.65).

**Figure 3:**
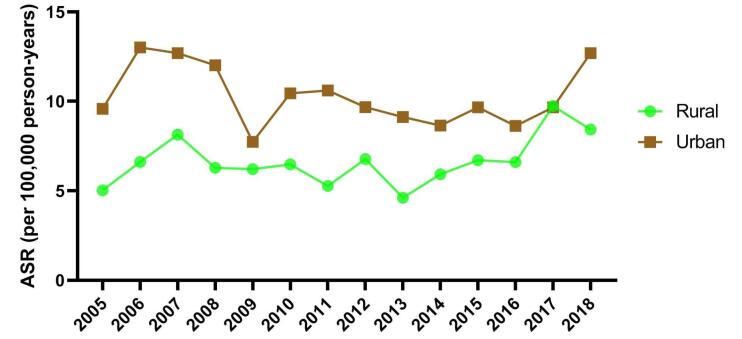
Time Trends of ASR (Per 100 000 Persons-Year) of Non-melanoma Skin Cancers by Area of Residence

 There was no significant difference in the time trend of incidence of NMSC between urban (EAPC = -1.05; 95% CI: -4.98 to 3.04) and rural (EAPC = 2.09; 95% CI: -2.95 to 7.38) residents (Figure 3).

 The number, crude rate, ASR, and 95% CI of ASR for melanoma and NMSCs by the city of residence are presented in Table 2. The rate of both melanoma and NMSCs was highest in the most populated cities of the province, i.e. Gorgan (capital city) and Gonbad (second most populated city).

**Table 2:** Number, Crude Rate, ASR, and 95% CI of ASR for Melanoma and NMSCs in Golestan, Iran, by City

**City of Residence**	**Non-melanoma**					**Melanoma**				
	**Number**	**Crude rate**	**ASR**	**ASR_L**	**ASR_U**	**Number**	**Crude rate**	**ASR**	**ASR_L**	**ASR_U**
Aliabad	112	5.99	8.01	6.48	9.54	5	0.27	0.39	0.04	0.74
Aqqalq	76	4.4	6.25	4.78	7.72	5	0.29	0.39	0.04	0.74
Azadshahr	60	4.6	6.6	4.88	8.32	1	0.08	0.09	0	0.27
Bandaregaz	47	7.26	6.54	4.64	8.44	3	0.46	0.4	0	0.85
Bandareturkmen	62	3.62	4.5	3.34	5.66	8	0.47	0.68	0.19	1.17
Galikesh	8	4.2	5.87	1.75	9.99	1	0.53	0.72	0	2.13
Gomishan	11	5.25	5.9	2.29	9.51	1	0.48	0.49	0	1.45
Gonbad	265	5.84	8.62	7.54	9.7	18	0.4	0.56	0.29	0.83
Gorgan	645	10.22	12.57	11.57	13.57	36	0.57	0.67	0.43	0.91
Kalaleh	66	3.06	5.45	4.08	6.82	9	0.42	0.48	0.17	0.79
Kordkuy	96	9.81	9.65	7.67	11.63	6	0.61	0.63	0.12	1.14
Maravetapeh	4	2.18	4.77	0	9.89	0	0	0	0	0
Minoodasht	86	5.07	6.66	5.21	8.11	8	0.47	0.59	0.16	1.02
Ramian	43	3.62	4.97	3.42	6.52	8	0.67	0.79	0.22	1.36

Tables 3 and 4 show the number, crude rate, ASR, and 95% CI of ASR for NMSCs by city, sex, and residence area. The occurrence of NMSC was found to be more prevalent in the western sections of the Golestan province, as opposed to the eastern parts.

**Table 3:** Number, Crude Rate, ASR, and 95% CI of ASR for NMSCs in Golestan, Iran, by City and Sex

**City of Residence**	**Male**					**Female**				
	**Number**	**Crude Rate**	**ASR**	**ASR_L**	**ASR_U**	**Number**	**Crude Rate**	**ASR**	**ASR_L**	**ASR_U**
Aliabad	68	7.28	9.66	7.25	12.07	44	4.7	6.48	4.52	8.44
Aqqalq	47	5.49	8.14	5.67	10.61	29	3.33	4.51	2.79	6.23
Azadshahr	35	5.38	7.61	5	10.22	25	3.83	5.47	3.22	7.72
Bandaregaz	32	9.96	8.91	5.73	12.09	15	4.61	4.29	2.09	6.49
Bandar Torkaman	39	4.55	6	4.04	7.96	23	2.69	3.1	1.79	4.41
Galikesh	5	5.22	7.87	0.83	14.91	3	3.17	3.9	0	8.37
Gomishan	8	7.54	9.19	2.37	16.01	3	2.89	3.12	0	6.67
Gonbad	164	7.23	10.97	9.21	12.73	101	4.45	6.41	5.12	7.7
Gorgan	385	12.1	15.23	13.66	16.8	260	8.31	9.97	8.72	11.22
Kalaleh	47	4.39	8.04	5.63	10.45	19	1.75	2.99	1.58	4.4
Kordkuy	61	12.41	12.09	8.93	15.25	35	7.18	7.19	4.76	9.62
Maravetapeh	4	4.35	9.77	0	20.39	0	0	0	0	0
Minoodasht	45	5.37	7.27	5.06	9.48	41	4.77	6.01	4.13	7.89
Ramian	25	4.24	6.4	3.79	9.01	18	3.02	3.63	1.89	5.37

**Table 4:** Number, Crude rate, ASR, and 95% CI of ASR for NMSCs in Golestan, Iran, by City and Residence Area

**City of Residence**	**Urban**					**Rural**				
	**Number**	**Crude Rate**	**ASR**	**ASR_L**	**ASR_U**	**Number**	**Crude Rate**	**ASR**	**ASR_L**	**ASR_U**
Aliabad	68	6.99	9.43	7.1	11.76	44	4.9	6.53	4.55	8.51
Aqqalq	20	3.73	6.5	3.52	9.48	56	4.71	6.3	4.58	8.02
Azadshahr	31	4.29	6.29	4	8.58	29	4.99	6.9	4.31	9.49
Bandaregaz	30	8.12	7.66	4.86	10.46	17	6.13	5.12	2.61	7.63
Bandar Torkaman	44	4.18	5.17	3.58	6.76	18	2.73	3.5	1.85	5.15
Galikesh	4	5.61	7.45	0.14	14.76	4	3.36	4.68	0.03	9.33
Gomishan	5	4.49	4.66	0.45	8.87	6	6.09	7.3	1.22	13.38
Gonbad	172	8.46	11.11	9.39	12.83	93	3.71	6.13	4.86	7.4
Gorgan	485	10.4	13.25	12.03	14.47	160	9.72	10.9	9.16	12.64
Kalaleh	19	3.44	6.08	3.22	8.94	47	2.93	5.29	3.7	6.88
Kordkuy	52	10.25	11.1	7.98	14.22	44	9.33	8.56	5.95	11.17
Maravetapeh	0	0	0	0	0	4	2.56	5.39	0	11.13
Minoodasht	39	6.19	9.35	6.25	12.45	47	4.41	5.44	3.83	7.05
Ramian	17	3.68	4.77	2.44	7.1	26	3.59	5.11	3.05	7.17

**Figure 4:**
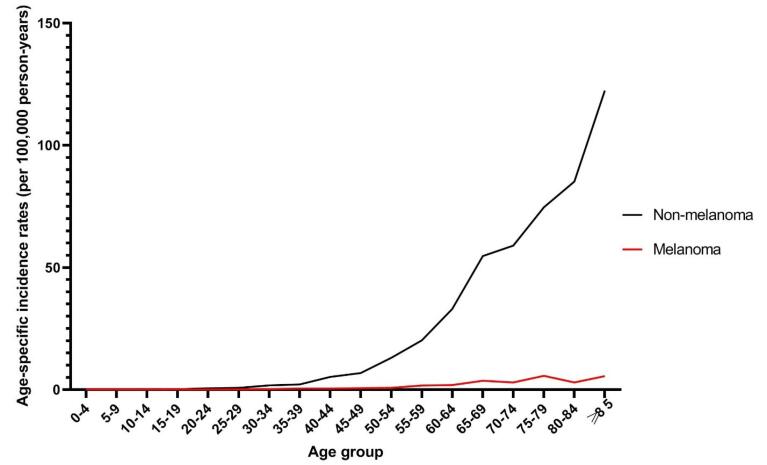
Trends in Age-Specific Incidence Rate (Per 100 000 Persons-Year) for Melanoma and Non-melanoma Skin Cancers

 The age-specific incidence rate for non-melanoma and melanoma skin cancers in the total population was highest in those aged ≥ 85 and 75-79 years, respectively (Figure 4). The age-specific incidence rate for NMSC was lowest among those aged 15-19 years, while melanoma was not detected in those aged 5-14 and 20-24 years. The age-specific incidence rates for both cancers increased by age regardless of gender. Melanoma was not detected in the younger male population, i.e. those aged 24 and younger. In residents of urban and rural areas, the age-specific incidence rate was highest in the elderly population for both skin cancers.

## Discussion

 The prevalence of skin cancer, encompassing melanoma and NMSC, is notably high among individuals aged 54 years and above, making it the predominant form of cancer within this demographic. Furthermore, in Iran, it ranks as the second most frequently diagnosed cancer, highlighting its significant impact on the population. In the Golestan province, skin cancer is the third most common cancer after breast and colorectal cancer.^21^ The present study was conducted to determine the incidence of and temporal changes in skin cancers in the Golestan province between 2005 and 2018. The incidence of skin cancer was investigated by gender, place of residence, cancer type, and method of diagnosis. Of 31 252 registered cancer cases in the province during the study period, 1690 cases (5.4%) were skin cancer.

 The ASR of NMSC was 8.49 (10.6 in men and 6.45 in women) in the 14-year study period and 10.82 (13 in men and 8.84 in women) in 2018. Moreover, the ASR of melanoma skin cancer was 0.56 during the 14-year study period and 0.4 in 2018.

 In a study by Kiani et al in the Fars Province of Iran, the ASR of skin cancer was reported as 13.05.^23^ In a study in the Isfahan province of Iran, the incidence of skin cancer in 2008 was 30.8 and 18.9 in men and women, respectively.^24^ A previous study in the Golestan province in 2004 reported the incidence of skin cancer at 13.23 in men.^22^ According to Razi et al, the rate of skin cancer in 2008 was highest in the Semnan Province with incidence rates of 22.62 and 15.77 for skin cancer in men and women, respectively.^7^ In 2014, Ghonche et al conducted a retrospective cross-sectional study to determine the epidemiology and changes in the incidence of skin cancer in southern Iran and reported incidence rates of 23.81 and 15.05 for men and women, respectively.^13^ Based on the data of the cancer registry center in Schleswig-Holstein, Germany, the ASR of NMSC in the Schleswig-Holstein region was 96 and 82.8 in men and women (with the world standard population), respectively.^25^

 According to the IARC, the ASR for NMSC in 2020 was estimated at 11 (men: 15.1, women: 7.9) worldwide, 1.5 (men: 1.8, women: 1.2) in Asia, and 4 (men: 5.5, women: 2.4) in Iran.^12^ Compared to the IARC estimate, the incidence of NMSC in our study was higher than the estimated rate for Iran and Asia and close to the total ASR in the world. In addition, the incidence of NMSC in men in our study was lower than the global rate and higher than the rate for Asia, while the incidence of NMSC in women was higher than the rate estimated for Asia and worldwide. The estimated incidence of NMSC in our study was lower than that of developed countries such as Australia (ASR = 140), New Zealand (ASR = 127.5), the United States (ASR = 64.9), and Canada (ASR = 60.6). Furthermore, the estimated incidence of NMSC in our study was higher than that of the national average (ASR = 4) and Iran’s neighboring countries such as Azerbaijan (ASR = 3.4), Pakistan (ASR = 2.8), Iraq (ASR = 1.5), and Saudi Arabia (ASR = 1.2). According to the IARC estimates, the ASR of melanoma was 3.4 worldwide (3.8 in men and 3 in women), 0.42 in Asia (0.46 in men 0.46 and 0.39 in women), and 0.67 in Iran (0.72 in men and 0.6 in women). The incidence of melanoma in our study was lower than the estimated incidence in the globe and Iran and more than the estimated incidence in Asia. Moreover, the incidence of melanoma in our study area was lower than the estimated incidence in developed countries such as Australia (ASR = 36.6), New Zealand (31.6), and Denmark (29.7) but higher than developing countries such as Jordan (0.37), Bangladesh (0.31), Pakistan (0.29), and Qatar (0.23).^26^

 The incidence rate of skin cancer in our study area was lower than the rates reported by studies in the central and southern parts of Iran. This could be due to the proximity of the southern areas of the country to the equator and the greater UV exposure, which increases the risk for skin cancer; in fact, the incidence of skin cancer doubles when the latitude decreases by nearly 10 degrees.^7^ In addition, most referral centers in the country are located in the central and southern provinces which have better diagnostic and treatment facilities. Other factors such as differences in the population structure, life expectancy, population of elderly people, socioeconomic status, and lifestyle may also affect the prevalence of skin cancer in this area.^13^ The difference in the incidence of skin cancer between our study area and developed countries could be attributed to the strong diagnostic facilities and screening programs in those countries, which highlights the need for the integration of skin cancer screening programs into the health system of Iran. In Germany, the NMSC screening program was initiated during 2003–2004 and its effect on the incidence of NMSC was investigated by Eisemann and colleagues. By using the national skin cancer screening program, an increase was observed in the incidence of NMSC in the Schleswig-Holstein region, as much as 47% in women and 34% in men, when comparing the pre-screening period (1998–2000) with the nationwide screening period (2010-2008).^25^ One of the main reasons for the difference in the incidence of skin cancer between Iran and other countries can be the difference in clothing and fashion. Other environmental factors include occupation, working hours, air humidity, smoking, and exposure to carcinogenic substances and infectious agents. Indeed, increasing public awareness about the risk factors of skin cancer and the need for using sunscreen and protective items such as hats and sunglasses could reduce the incidence of skin cancer.

 In this study, both forms of skin cancer were more common in men (60.1%), and the men: women ratio was 1.56, which is in line with the reports of the IARC^26^ and previous studies.^6,7,9,13,21,23,25,27^ Apart from the issue of hijab in women which hinders sunlight exposure, the higher incidence of skin cancer in men could be related to the higher rate of outdoor occupations in men and the subsequent exposure to sunlight and other carcinogenic factors.^3,27^

 In our study, the highest and lowest ASR of skin cancer was observed in people aged ≥ 85 years for non-melanoma and melanoma, 0-4 years for non-melanoma, and 5-14 years for melanoma. In a previous study in the Golestan province in 2004, which was conducted only on men, the rate of skin cancer was highest in subjects aged 80-84 years old.^22^ In other studies, the incidence rates were highest in the middle-aged and elderly.^11,21,23,24,27^ In a study in Kermanshah between 2003 and 2018, the incidence of skin cancer was highest in the seventh decade of life.^6^ A study in Canada showed that the incidence of skin cancer was higher in older people compared to younger people.^28^ In a study on 209 countries of the world in 2019, the rate of skin cancer was higher in older people, and an increasing trend of cancer was observed especially for areas that encompass people aged 55 years.^29^ In a 30-year study in Hong Kong, demographic factors were reported to be pivotal in the rising incidence of NMSC in Hong Kong. Moreover, an increase in the incidence of NMSC in people aged 60 years and older was anticipated, which would increase the NMSC burden in the country.^30^ In our study, the incidence of skin cancers in the older subjects was higher compared with other studies in the same area. This can be mainly attributed to the changes in the socioeconomic status and rise of the elderly population.

 In our study, the ASR for NMSC increased from 7.17 in 100 000 people in 2005 to 10.82 in 100 000 in 2018. In addition, this increase in ASR was more profound in men (from 7.04 to 13 in 100 000) compared to women (from 7.14 to 8.84 in 100 000). Overall, the trend analysis during the 14-year period shows a relative increase in the incidence of NMSC. Most studies conducted in different parts of Iran have also shown the increasing trend of NMSC in recent years^7,11,13,23,31^ and the higher overall incidence rate in men.^7,32^ Despite the increasing trend of NMSC in most parts of Iran, a study based on the cancer registration statistics of Iran reported a decreasing trend for skin cancer during 2000-2016, making skin cancer the second most common cancer in Iran. According to this study, the ASR of NMSC in women and men decreased from 13.97 and 19.96 in 2000 to 13.1 and 15.5, respectively.^21^ The decreasing trend of skin cancer was also reported by a study in the Sistan and Baluchestan Province in 2016.^33^ Nevertheless, the results of studies in most parts of the world including Germany,^34,35^ Brazil,^36,37^ Canada,^28,38^ Spain^39^ indicate the increasing trend of NMSC. For instance, in the study by Leiter et al, the incidence of NMSC in Germany increased 10–22 fold between 1970 and 2012, while the associated mortality rate had a decreasing trend.^34^

 In the study by Abbas et al, the incidence of NMSC increased in Canada from 1960 to 2015, while the rate in the younger population had a slow decreasing trend.^28^ Another study in Canada also reported an overall increase in the incidence of skin cancers between 2000 and 2010 38. In 2016, a study reported 6.1 and 5.1% increases in the annual incidence of SCC and BCC, respectively.^39^

 In a study in Hong Kong, the ASR of NMSC was relatively stable in men and decreased in women during 1990-2019, but the prevalence of NMSC was still increasing. The study predicted an increasing trend during 2019-2030, particularly in the elderly population, probably because of raised awareness of the general population and physicians about skin cancer and increased availability and accuracy of diagnostic tools.^30^ In Wales, the rate of NMSC increased by 7% during 2016-2019, making this malignancy the most common cancer in the country.^40^

 In the study by Zhang et al on the trends and statistics of skin cancer in 209 countries between 1990 and 2019, the rate of all three types of skin cancer increased in the 21 geographical regions under study.^29^ According to this study, the rate of melanoma, SCC, and BCC increased in 167, 204, and 110 countries, respectively. Similar to the results of most previous studies, our findings indicated a relative increase in the incidence of skin cancer in the Golestan province during the 14-year period. However, the actual statistics and the increasing trend may be higher than the rate reported in our study since the focus of the health system of Iran has been on the screening of other cancers such as breast cancer or colorectal cancer and there is currently no screening program for skin cancer in the country. In this regard, it should be noted that NMSC lesions are sometimes operated by general practitioners rather than dermatologists, especially in low socioeconomic areas, which may result in the misdiagnosis of the lesions as simple moles that are not subjected to pathological examination. Indeed, initiating skin cancer screening programs, training general practitioners, raising public awareness, and launching an NMSC cancer registry can significantly contribute to the timely diagnosis and treatment of skin cancers.

 In our study, the incidence of NMSC was significantly higher in urban areas than rural areas. This finding is in line with the results of studies conducted in Iran^27^ and Ireland.^41^ According to the report of the Welsh Cancer Intelligence and Surveillance Unit in March 2023, NMSC is 26% more prevalent in people living in more affluent areas with a higher level of public health than in those living in deprived areas with a lower level of health.^40^ The study by Zhang involving 209 countries also indicated that the ASR of all skin cancer types is higher in people with a higher socioeconomic status.^29^ For instance, the overall ASR of melanoma was 3.56 around the globe and 12.40, 0.7, and 0.51 in areas with high, middle, and low socioeconomic status, respectively.

 The level of education and awareness of people, access to diagnostic and treatment facilities, and a higher socioeconomic status in more privileged areas make people more sensitive and persistent about health problems and more expectant of healthcare services.^41^ In addition, the higher incidence of skin cancer in cities could be attributed to the difference in risk factors, occupational activities, and clothing between people living in rural and urban areas. In our study, the incidence of NMSC was higher in the western areas of the Golestan province where people have a higher sociodemographic status. Lifestyle and outdoor activities, dietary habits, residential proximity to chemical pollutants, awareness about skin cancer and its symptoms, and easy access to dermatologists and healthcare facilities may contribute to the higher incidence of skin cancer among the urban population.

 This epidemiological study was the first to investigate the trend of skin cancer in the Golestan province, which can be a cornerstone for examining more details and indicators such as mortality, recurrence, etc. in future studies.

 One of the strengths of this study was the retrieval of data from the GPCR, which is both reliable and valid. It was not possible to check the subjects’ socio-economic status, including income, marital status, education level, or employment status, which could be a limitation of the present study. Furthermore, there was no data on the pathological type of NMSC skin cancers, outcomes, and recurrence.

## Conclusion

 The findings of our study indicate that the ASR of skin cancer (melanoma and non-melanoma) increased relatively during 2005-2018. In addition, the ASR of NMSC was significantly higher in men, urban residents, and western parts of the province. Considering the high level of sunlight exposure in childhood and adolescence, it is suggested to incorporate educational and prevention programs to address this issue in the schools’ agenda. For early detection of skin cancer, it is recommended to plan for screening programs, raise awareness in the general population, and train healthcare providers on the symptoms of skin cancer. It is also suggested to evaluate the relationship of ethnic diversity with the incidence of skin cancer and its mortality rate in the Golestan province.
